# Differential response in patients with large cell neuroendocrine carcinoma of the lung to initial therapy: A case series

**DOI:** 10.1002/cnr2.1754

**Published:** 2022-11-11

**Authors:** Yutaka Takahara, Takuya Tanaka, Yoko Ishige, Ikuyo Shionoya, Kouichi Yamamura, Takashi Sakuma, Kazuaki Nishiki, Keisuke Nakase, Masafumi Nojiri, Ryo Kato, Shohei Shinomiya, Taku Oikawa, Shiro Mizuno

**Affiliations:** ^1^ Department of Respiratory Medicine Kanazawa Medical University Kahoku‐gun Ishikawa Japan

**Keywords:** chemotherapy, large cell neuroendocrine carcinoma, non‐small cell lung cancer, small cell lung carcinoma, treatment

## Abstract

**Background:**

Large cell neuroendocrine tumors of the lung (LCNEC) are rare. Chemotherapy with the small cell lung carcinoma (SCLC) regimen is the most appropriate treatment for LCNEC. However, there is evidence that the non‐small cell lung cancer regimen is also effective in some reported cases. Due to the differences in response to LCNEC treatment, a standard of care for LCNEC has not been established.

**Cases:**

The clinical records of nine patients with LCNEC who were treated with anticancer drugs based on an SCLC regimen from March 2016 to March 2022 were retrospectively reviewed. The patients who responded to treatment after one cycle of systemic chemotherapy were compared to those who did not respond. All patients in the responder group had a performance status (PS) of 0 or 1. However, 5 of the 6 patients in the non‐responder group had a PS of 2 or 3, indicating that many patients were in poor general condition. Although patients with multiple metastases to more than one organ prior to treatment were not identified in the responder group, five of these patients were in the non‐responder group.

In the non‐responder group, all patients discontinued treatment due to deterioration of general condition during first‐line treatment. Thus, none of them were able to start the second‐line treatment.

**Conclusion:**

The results of this study may suggest that early diagnosis and initiation of treatment before multiple organ metastasis development and PS decline may have clinical implications that could lead to improved treatment response in patients with LCNEC.

## 1 INTRODUCTION

Large cell neuroendocrine tumors of the lung (LCNEC) are relatively rare, accounting for approximately 3% of all lung cancers.^1^ LCNEC was previously classified under non‐small cell lung cancer (NSCLC) as a subtype of large cell carcinoma.^2^ However, as a neuroendocrine tumor with lung carcinoid, following the 2015 revision of the WHO classification, LCNEC was reclassified as small cell lung cancer (SCLC).^3^ Owing to its rarity, there is no standard treatment of LCNEC yet.^4^ According to the American Society of Clinical Oncology guidelines, the platinum‐etoposide chemotherapy (SCLC‐PE) regimen is considered the most appropriate treatment^5^ and is applied after diagnosis. However, the efficacy of this regimen is insufficient.^6^ In addition, there have been reports that NSCLC regimens were effective in the treatment of LCNEC at an advanced stage. Thus, no consensus has been reached on the most appropriate treatment for LCNEC patients.^7^ Although the clinical characteristics of LCNEC are generally associated with high metastatic and low survival rates, a wide range of survival rates have been reported.^8^ It has been suggested that these differences in treatment response and clinical characteristics might arise from biologically heterogeneous subgroups of LCNEC.^8^

Recently, attempts have been made to classify LCNEC based on its molecular subtypes, which can be used for tailoring personalized therapy.^4^ However, it is currently difficult to confirm these subtypes before treatment in actual clinical practice. Therefore, the SCLC initial treatment remains the rational approach for patients with LCNEC. However, if the SCLC regimen is continued in a group of patients who effectively respond to the NSCLC regimen, the side effects are expected to worsen their systemic condition and decrease their survival rate.^9^ We focused on whether it was possible to identify patients with LCNEC who would respond to SCLC‐based chemotherapy before treatment.

This study summarizes the clinical findings of nine patients with LCNEC who were initially treated with SCLC‐based therapy and their clinical characteristics, laboratory data, and response to treatment.

## 2 CASES

### 2.1 Methods

Cases of advanced‐stage (stage IV, recurrent) LCNEC treated with anticancer agents with SCLC regimens as first‐line therapy from March 2016 to March 2022 were retrospectively recruited.

Data on age, gender, smoking history, performance status (PS), tumor proportion score (TPS), tumor markers, and treatment details were collected. Computed tomography (CT) was performed to determine treatment efficacy: chest CT was performed after one cycle of chemotherapy.

Responses were evaluated based on the Response Evaluation Criteria in Solid Tumors version 1.1. Patients with complete response (CR) and partial response (PR) were allocated to the responder group, and those with stable (SD) and progressive disease (PD) were included in the non‐responder group. The clinical data of the two groups were compared.

The study was conducted by the provisions of the Declaration of Helsinki and was approved by the Ethics Committee of Kanazawa Medical University Hospital (Approval No. I722).

## 3 HISTOLOGICAL CLASSIFICATION

Histological diagnosis of the tumor was made by a pathologist according to the 2015 World Health Organization Classification of Lung and Pleural Tumors criteria.^3^

Immunohistochemistry was performed using the PD‐L1 kit (PD‐L1 IHC 22C3 pharmDX; Dako) according to the manufacturer's instructions.^10^ The TPS was used to classify the expression status as follows: <50% (Low) and >50% (High).

## 4 PATIENT BACKGROUND

A total of nine cases were identified in this study. Patient characteristics are shown in Table 1. Three patients were enrolled in the responder group and six in the non‐responder group. In the responder group, all patients had an Eastern Cooperative Oncology Group (ECOG) PS of 0 or 1. However, five patients in the non‐responder group had a PS of 2 or 3. The mean number of cycles of initial chemotherapy in the responder and non‐responder groups was 5.3 and 1.8, respectively. Although patients with multiple metastases in more than one organ prior to treatment were not identified in the responder group, five were found in the non‐responder group.

**TABLE 1:** Patient characteristics

	Responder	Non‐responder
Total, *n*	3	6
Age, years (range)	68.0 (59–76)	69.3 (59–75)
Sex (Male/Female)	(3/0)	(3/3)
Smoking history (never/prior·current)	(0/3)	(0/6)
ECOG PS (0–1/2–4)	(3/0)	(1/5)
Stage IV disease (de novo/recurrent)	(1/2)	(6/0)
Initial treatment cycles	5.3 (4–6)	1.8 (1–3)
CEA (ng/ml)	145.1 (5.0–407.0)	192.2 (2.0–1126.0)
(≦5.0/>5.0)	(2/1)	(4/2)
Pro‐GRP (ng/ml)	198.8 (62.4–431.0)	493.5 (18.0–1470.0)
(≦81.0/>81.0)	(2/1)	(3/3)
NSE (ng/ml)	13.1 (11.4–16.2)	116.7 (9.3–351.0)
(≦16.3/>16.3)	(0/3)	(3/1)
Distant metastasis		
Brain metastasis (yes/no)	(0/3)	(4/2)
Distant metastasis in 2 organs (yes/no)	(0/3)	(5/1)
PD‐L1 expression (22C3) (low/high/untested)	(1/0/2)	(4/0/2)

Abbreviations: CEA, carcinoembryonic antigen; ECOG PS, Eastern Cooperative Oncology Group Performance Status; NSE, neuron‐specific enolase; Pro‐GRP, pro‐gastrin‐releasing peptide; Recurrent, denotes patients who were diagnosed with Stage I/II at initial diagnosis, underwent surgical resection, and then recurred.

Table 2 shows the treatment details and their courses in the patients. The systemic chemotherapy regimen was selected at the discretion of the attending physician. Eight of the nine patients were administered carboplatin (CBDCA) + etoposide (VP‐16) as initial therapy. One patient in the non‐responder group (case 7) received CBDCA + CPT‐11 as first‐line therapy.

**TABLE 2:** Clinical characteristics and course of the patients

Group	Case	Age/sex	PS	cStage	CEA	CYFRA	Pro‐GRP	NSE	TPS	Diagnosis (procedure)	Metastatic site	First‐line treatment (cycle)	After first‐line treatment (cycle)
R	1	59/M	1	cT3N2M1a	23.2	4.3	103	16.2	Untested	Pleural tumor (surgical)	Pleura	CBDCA + VP‐16 (6)	DTX + RAM (2)
R	2	76/M	0	Recurrent	407	6.1	62.4	11.4	5%	Lung tumor (surgical)	Lymph nodes	CBDCA + VP‐16 (6)	Nivolumab (3) → NGT (3) → PEM (1)
R	3	69/M	1	Recurrent	5	1.7	431	11.6	Untested	Lung tumor (surgical)	Pleura	CBDCA + VP‐16 (4)	None
Non R	4	70/F	1	cT4N3M1b	1126	2.5	1180	24.6	10%	Lung tumor (autopsy)	Lung, brain	CBDCA + VP‐16 (2)	None
Non R	5	67/F	2	cT1N3M1b	14.9	10.1	1470	81.9	0%	Lymph node (surgical)	Lung, brain	CBDCA + VP‐16 (3)	None
Non R	6	71/M	2	cT4N3M1b	2	2	39.8	Untested	0%	Pleural tumor (biopsy)	Pleura, bone	CBDCA + VP‐16 (1)	None
Non R	7	59/M	3	cT4N3M1b	1.7	11	18	Untested	Untested	Brain tumor (surgical)	Pleura, brain	CBDCA + CPT‐11 (2)	None
Non R	8	74/M	3	cT4N3M1b	2.3	65	65	351	Untested	Lung tumor (autopsy)	Pleura, peritoneum	CBDCA + VP‐16 (1)	None
Non R	9	75/F	2	cT2N1M1a	6.3	5.4	207	9.3	0%	Brain tumor (surgical)	Brain	CBDCA + VP‐16 (2)	None

Abbreviations: Autopsy, diagnosis by autopsy; CBDCA, carboplatin; CPT‐11, irinotecan; DTX, docetaxel; F, female; M, male; NGT, nogitecan; non‐R, non‐responder group; PEM, pemetrexed; Procedure, diagnosis procedure; PS, performance status; R, responder group; RAM, ramucirumab; Surgical, surgical biopsy; TPS, tumor proportion score; VP‐16, etoposide.

**FIGURE 1:**
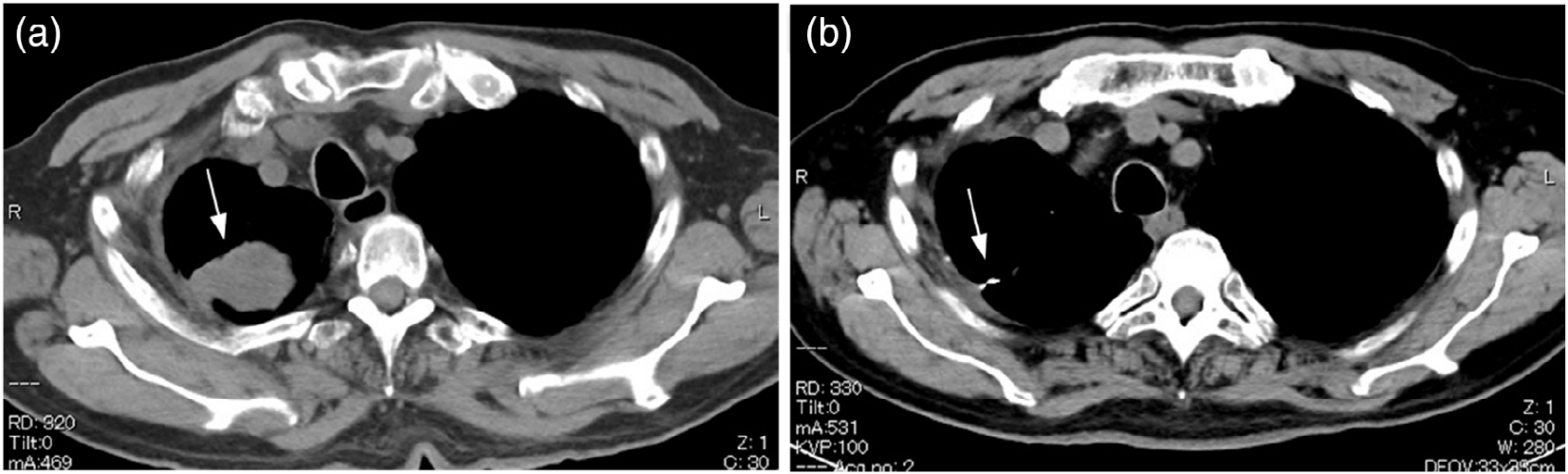
Computed tomography (CT) image of the chest (soft‐tissue windows). Chest CT image of a 69‐year‐old man (case 4 in Table 2). (A) Pre‐treatment chest CT shows a mass protruding from the right pleura (white arrow). (B) Chest CT after 1 cycle of treatment with CBDCA + VP‐16 therapy shows disappearance of the right pleural mass (white arrow)

In the responder group, all patients completed first‐line therapy, and one patient (case 3) achieved CR (Figure 1). In the non‐responder group, all patients who received the first‐line treatment group presented with SD, and none experienced PD. All patients were unable to complete the first‐line treatment due to deterioration of their general condition, hence none of them could start the second‐line treatment. Representative cases in the responder and non‐responder groups are presented below.

### 4.1 Presentation of cases

#### 4.1.1 The responder group

The patient was a 69‐year‐old man (case 3 in Table 2). He was diagnosed with LCNEC (pT1bN0M0 pStage IA2) after partial right upper lobectomy and was followed up without adjuvant chemotherapy. He was a current smoker of 15 cigarettes per day with a 48‐year history of smoking. Although the patient had a history of manic‐depressive episodes, he received no medical treatment at the time of presentation. He reported mild chest pain in the right anterior thoracic region starting approximately 6 months postoperatively. Therefore, a chest CT was performed and showed a recurrent tumor in the right pleura (Figure 1A).

**FIGURE 2:**
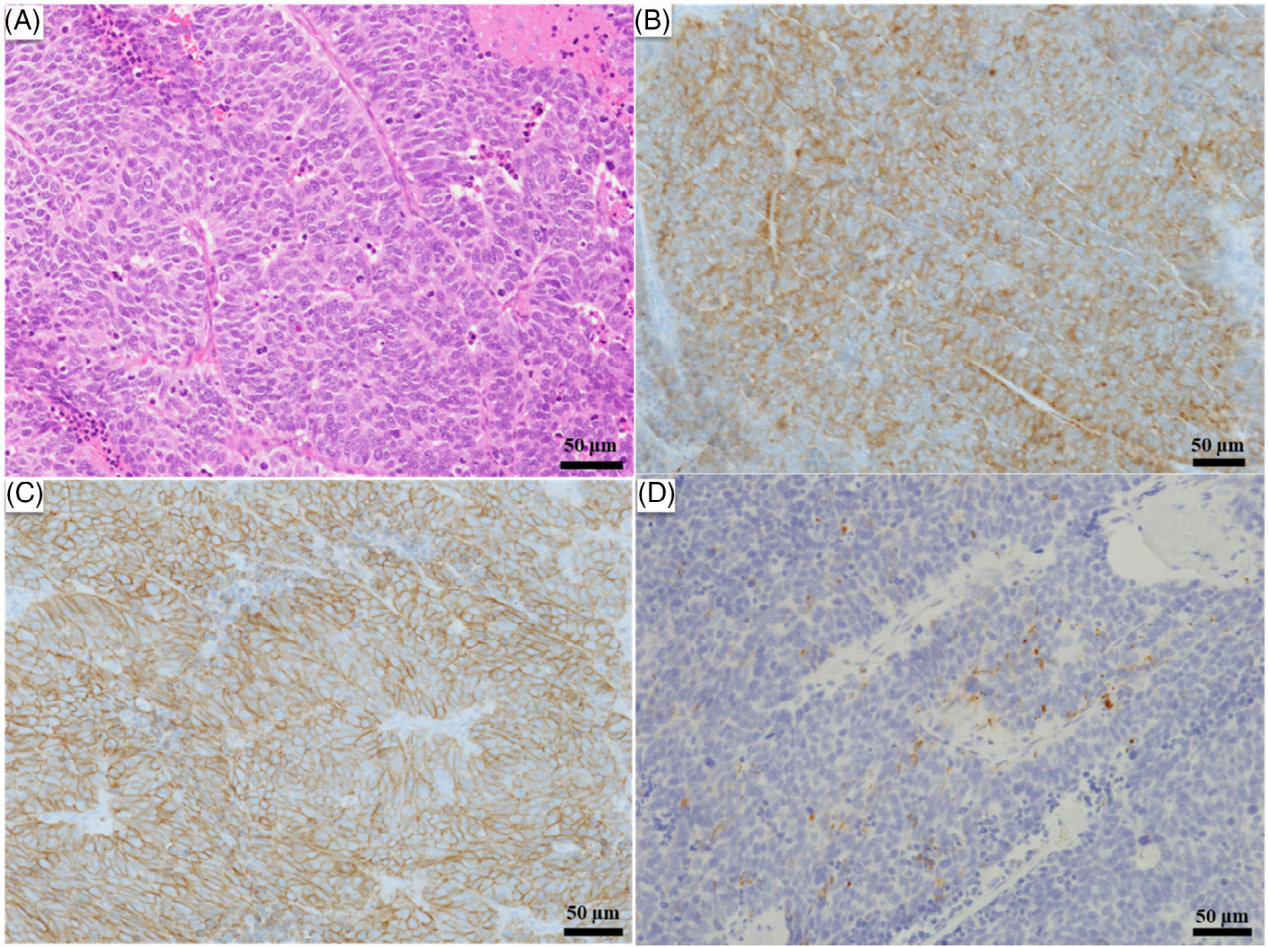
Histological and immunohistochemical findings. Histopathology of the lung tumor (hematoxylin and eosin [H&E] staining) shows large tumor cells with numerous mitotic figures and a rosette‐like arrangement. Immunostaining shows positivity for CD56 and synaptophysin and partial positivity for chromogranin A. (A) H&E staining, bar: 50 μm. (B) Synaptophysin staining, bar: 50 μm. (C) CD56 staining, bar: 50 μm. (D) Chromogranin A staining, bar: 50 μm. (A–D) Magnification, ×20

Histopathology of the lung tumor (hematoxylin and eosin [H&E] staining) showed large tumor cells with multiple mitotic figures and a rosette‐like arrangement. Regarding immunostaining, the tumor cells were positive for CD56 and synaptophysin, and partially positive for chromogranin A, consistent with LCNEC (Figure 2).

Chemotherapy with CBDCA + VP‐16 was administered, and chest CT confirmed that CR was achieved after one cycle of treatment (Figure 1B). He completed a total of four cycles of treatment, resulting in disease control and no recurrence.

#### 4.1.2 The non‐responder group

**FIGURE 3:**
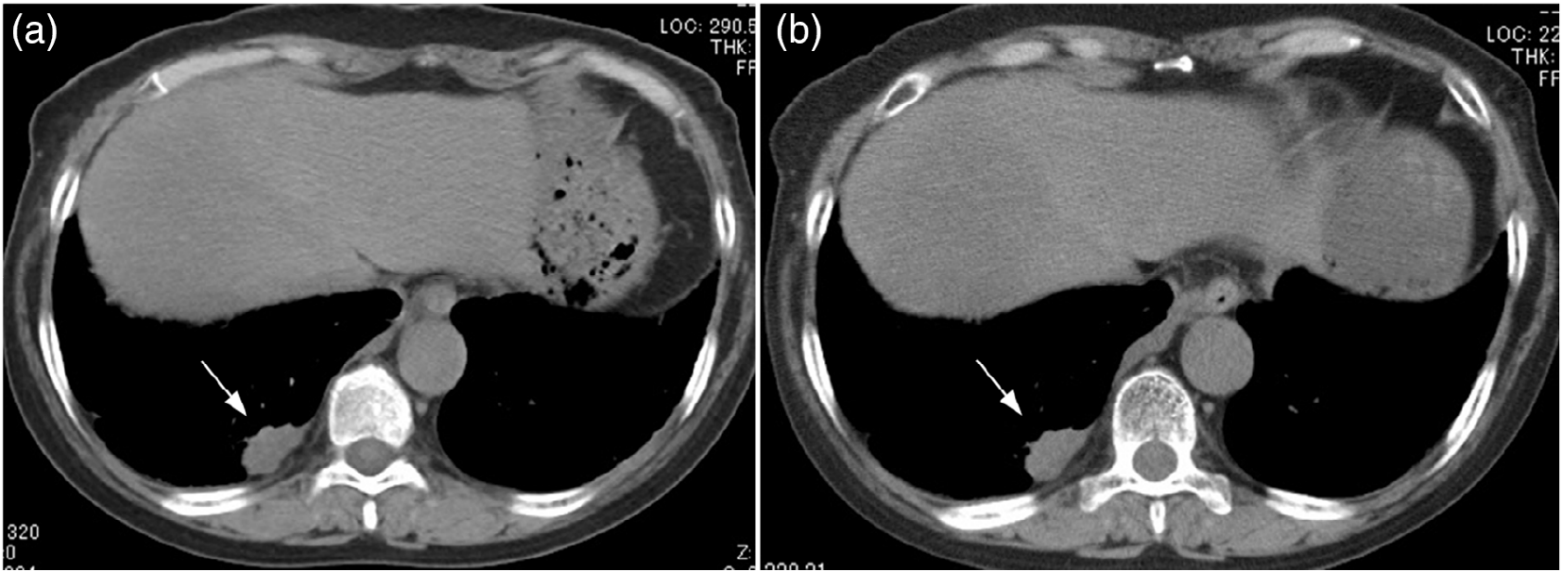
CT image of the chest (soft‐tissue windows). Chest CT image of a 67‐year‐old woman (case 5 in Table 2). (A) Pretreatment chest CT shows a 23 mm × 10 mm mass in the right lower lobe (white arrow). (B) Chest CT after one cycle of CBDCA + VP‐16 therapy shows no change in the size of the right lower lobe tumor (white arrow)

The patient was a 67‐year‐old woman (case 5 in Table 2). She presented to our clinic with the chief complaint of progressive dyspnea and anorexia that began 1 month prior to her visit. She was a current smoker of 20 cigarettes per day, with a 48‐year history of smoking. A chest CT scan at the time of presentation revealed a right lower lobe tumor (Figure 3A). Brain magnetic resonance imaging showed multiple brain metastases. A biopsy of the right supraclavicular lymph node was performed.

**FIGURE 4:**
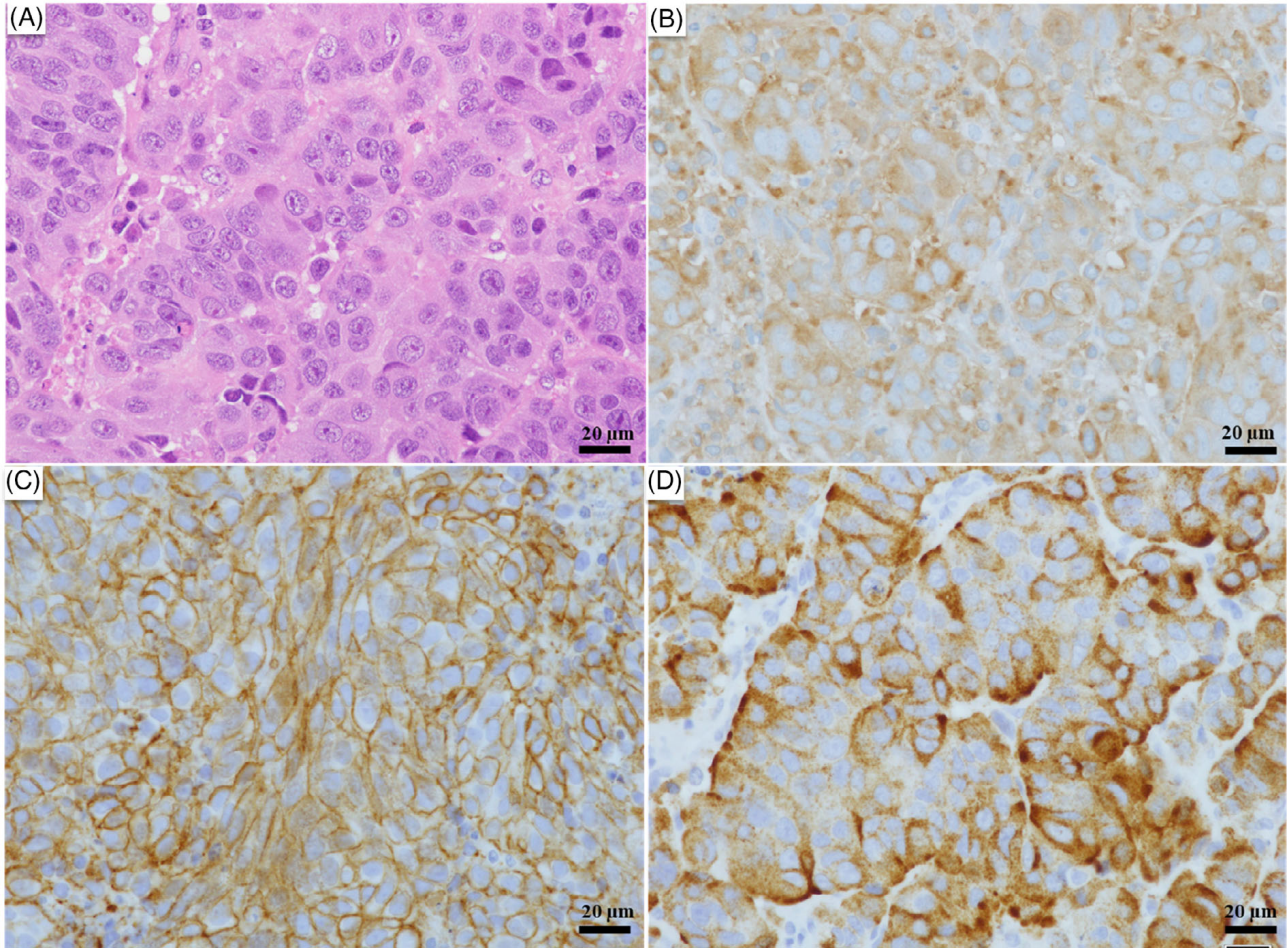
Histological and immunohistochemical findings. Histopathology of the lymph node (hematoxylin and eosin [H&E] staining) shows a palisade of tumor cells with a high nuclear/cytoplasmic (N/C) ratio and nuclear fission. Immunostaining reveals positivity for CD56, synaptophysin, and chromogranin A. (A) H&E staining, bar: 20 μm. (B) Synaptophysin staining, bar: 20 μm. (C) CD56 staining, bar: 20 μm. (D) Chromogranin A staining, bar: 20 μm. (A–D) Magnification, ×40

Histopathology (H&E staining) of the lymph node showed a palisade of tumor cells with a high nuclear/cytoplasmic (N/C) ratio and nuclear fission. After immunostaining, the tumor cells were positive for CD56, synaptophysin, and chromogranin A, consistent with LCNEC (Figure 4). The patient was treated with chemotherapy with CBDCA + VP‐16 as initial therapy. After one cycle of treatment, chest CT showed no change in tumor size, and SD was achieved (Figure 3B). Regarding brain metastases, whole‐brain irradiation of 37.5 Gy (2.5 Gy/day, 15 times) was performed. Despite three cycles of systemic chemotherapy, the tumor showed an increasing trend, and aggressive treatment was terminated due to the decline in PS. The patient died of cancer approximately 5 months after treatment initiation.

## 5 DISCUSSION

Various results have been reported on the chemosensitivity in patients with LCNEC to SCLC‐PE, and no consensus has been reached on whether LCNEC should be managed clinically as SCLC or NSCLC.^8^ These differences in treatment response were attributed to the heterogeneous cellular morphology and biology of LCNEC.^11^

Recent detailed genomic analysis of LCNEC has identified two molecular subtypes of LCNEC: NSCLC‐like and SCLC‐like.^8^, ^12^, ^13^ It has been suggested that SCLC‐like LCNEC responds to the SCLC regimen of chemotherapy and NSCLC‐like LCNEC responds better to the NSCLC regimen of chemotherapy rather than SCLC‐PE.^6^, ^14^, ^15^ However, confirmation of molecular subtypes prior to treatment is not approved within the current insurance coverage in Japan. Furthermore, in patients with advanced LCNEC, tumor tissue is often inadequate for next‐generation sequencing after histopathological diagnosis.^16^ Therefore, this study aimed to determine the clinical characteristics of a group of patients with LCNEC who were expected to respond to treatment.

In this study, many cases with multiple organ metastases in two or more locations and poor PS were observed in patients in the non‐responder group. Hence, earlier diagnosis and initiation of treatment before the development of multiple organ metastasis or decline in PS may lead to increased survival rates among patients with LCNEC. In addition, in the non‐responder group, all patients presented with SD after one cycle of systemic chemotherapy. Although there was no disease progression, there was an early deterioration of the general condition, and primary treatment was not completed. In patients with SCLC, even PS3 may be a target for active treatment due to the high sensitivity to drug therapy and the possibility of PS improvement in response to treatment.^17^, ^18^

Among the studied patients, two (cases 7 and 8) were treated with pharmacological therapy in the hope that the treatment would improve PS, although they had PS3 and poor general condition. However, completion of first‐line treatment was not possible due to the deterioration of their general condition. It was suggested that drug therapy in patients with LCNEC might be less effective in improving PS compared to SCLC.

This study has several limitations. First, this was a single‐center study, and only nine LCNEC patients received the SCLC regimen of chemotherapy as initial therapy, with three patients in the response group, a very small number. The non‐responder group had more patients with multiple organ metastases and poor general conditions, which may have affected the response rate. Second, the response to the NSCLC regimen of chemotherapy could not be studied because all patients in the non‐responder group were unable to start the second‐line therapy. It is unclear whether the non‐responders were a subtype of LCNEC that responded to the NSCLC regimen of chemotherapy or a group of patients who had a poor response to drug therapy because of poor general health. In the future, analyses with a larger number of patients from multiple facilities are needed.

The results of this study may be useful for predicting pre‐treatment response to chemotherapy of SCLC regimens in patients with LCNEC for whom molecular subtyping is not available, as well as in predicting the subsequent course based on response to first‐line therapy. We believe that the results of this study provide important clinical implications for physicians currently treating cancer in clinical practice.

## 6 CONCLUSION

It was suggested that patients with LCNEC who were in good general condition before treatment and did not have multiorgan metastasis might respond to systemic chemotherapy with the SCLC regimen. In addition, patients with LCNEC who did not respond well after completing one cycle of systemic chemotherapy might experience early systemic deterioration, making even the completion of primary therapy difficult. Clinicians should be mindful of prompt test planning and early treatment in the care of patients with LCNEC. Early diagnosis and initiation of treatment before multiple organ metastasis development and PS decline may lead to improved treatment efficacy in patients with LCNEC.

## AUTHOR CONTRIBUTIONS

**Yutaka Takahara:** Conceptualization (lead); data curation (equal); formal analysis (equal); investigation (equal); methodology (lead); project administration (equal); writing – original draft (lead); writing – review and editing (lead). **Takuya Tanaka:** Data curation (supporting); writing – original draft (supporting). **Yoko Ishige:** Data curation (supporting); writing – original draft (supporting). **Ikuyo Shionoya:** Data curation (supporting); writing – original draft (supporting). **Kouichi Yamamura:** Data curation (supporting); writing – original draft (supporting). **Takashi Sakuma:** Data curation (supporting); writing – original draft (supporting). **Kazuaki Nishiki:** Data curation (lead); writing – original draft (supporting); writing – review and editing (supporting). **Keisuke Nakase:** Data curation (lead); writing – original draft (supporting). **Masafumi Nojiri:** Data curation (lead); writing – original draft (supporting). **Ryo Kato:** Data curation (supporting); writing – original draft (supporting). **Shohei Shinomiya:** Data curation (lead); writing – original draft (supporting). **Taku Oikawa:** Data curation (lead); writing – original draft (supporting). **Shiro Mizuno:** Project administration (supporting); writing – original draft (supporting); writing – review and editing (supporting).

## CONFLICT OF INTEREST

The authors have stated explicitly that there are no conflicts of interest in connection with this article.

## ETHICS STATEMENT

This study was approved by the Institutional Review Board of Kanazawa Medical University (Approval No. I722).

## CONSENT TO PARTICIPATE

In keeping with the policies for a retrospective review of Kanazawa Medical University, informed consent was not required.

## CONSENT FOR PUBLICATION

Written informed consent for publication of their case histories and associated images was obtained from the nine patients. A copy of the written consent is available for review by the Editor‐in‐chief of this journal.

## Data Availability

The data are available from the corresponding author on reasonable request.
